# New perspectives in fire management in South American savannas: The importance of intercultural governance

**DOI:** 10.1007/s13280-018-1054-7

**Published:** 2018-05-11

**Authors:** Jayalaxshmi Mistry, Isabel Belloni Schmidt, Ludivine Eloy, Bibiana Bilbao

**Affiliations:** 1 Department of Geography, Royal Holloway University of London, Egham, Surrey TW200EX UK; 2 Departamento de Ecologia, Universidade de Brasília, P.O. Box 04457, Brasília, DF CEP 70910-900 Brazil; 3 National Center for Scientific Research (CNRS), UMR 5281 ART-DEV, Paul Valéry University, 34090 Montpellier, France; 4 Centro de Desenvolvimento Sustentável, Campus Universitário Darcy Ribeiro Gleba A Universidade de Brasília - Asa Norte, Brasília, DF 70910-900 Brazil; 5 Departamento de Estudios Ambientales, Universidad Simón Bolívar, Apartado 89000, Caracas, 1080 Venezuela

**Keywords:** Brazil, Fire policy, Indigenous, Savanna, Traditional knowledge, Venezuela

## Abstract

Wildfires continue to cause damage to property, livelihoods and environments around the world. Acknowledging that dealing with wildfires has to go beyond fire-fighting, governments in countries with fire-prone ecosystems have begun to recognize the multiple perspectives of landscape burning and the need to engage with local communities and their practices. In this perspective, we outline the experiences of Brazil and Venezuela, two countries where fire management has been highly contested, but where there have been recent advances in fire management approaches. Success of these new initiatives have been measured by the reduction in wildfire extent through prescribed burning, and the opening of a dialogue on fire management between government agencies and local communities. Yet, it is clear that further developments in community participation need to take place in order to avoid the appropriation of local knowledge systems by institutions, and to better reflect more equitable fire governance.

## The burning issue

Wildfires wreak havoc on habitats and peoples around the world. The 2017 Chile wildfires, 2016 Fort McMurray fires in Canada, the regular catastrophic bushfires in Australia, Portugal and the USA, and the annual burning of vast tracts of forest and savanna ecosystems in the Amazon Basin and Indonesia are emblematic of this capacity for impact. Over the decades, scientists have expanded our understanding of fire behaviour and ecology, the effects of burning on landscape dynamics, soils and biodiversity, and fire’s contribution to global warming (Scott et al. 2014, 2016). Yet, the extensive occurrence of wildfires continues to highlight the gap between fire policies largely conceived in classic conservation terms within colonial histories, and local burning practices situated in specific environmental contexts (Eloy et al. 2018).

At the same time, there is mounting evidence to show the critical role of indigenous and traditional communities in effective fire management (Trauernicht et al. 2015). For example, satellite imagery from northern South America suggests that indigenous lands have lower incidence of wildfires and deforestation rates, which significantly contribute to maintaining carbon stocks and biodiversity (Nepstad et al. 2006; Nelson and Chomitz 2011; Flantua et al. 2013; Nolte et al. 2013; Welch et al. 2013; Walker et al. 2015). However, traditional ecological knowledge (TEK) on fire management is still poorly described, rarely addressing the spatial and seasonal patterns of local burning practices within the landscape. With the now widespread recognition that eliminating landscape fires is not only ecologically, but also socially and economically unviable in fire-prone ecosystems (Bilbao et al. 2010; Durigan and Ratter 2016; Mistry et al. 2016), countries in South America are moving towards the potential of an ‘intercultural fire governance’ (Rodríguez et al. 2013a, b); governance that acknowledges the multiple perspectives of landscape burning, thus reducing conflict amongst stakeholders, and supporting locally threatened biological and cultural diversity.

## From zero fire to prescribed burning

Fire has been used as a management tool by traditional communities in savanna and forest environments around the world for millennia (Bowman et al. 2011) and some ecosystems such as tropical savannas are dependent on regular burning (Durigan and Ratter 2016; de Carvalho and Mustin 2017). Nevertheless, most countries adopted ‘zero-fire’ policies intended to avoid and control virtually any fires, by focusing on fire-fighting techniques such as fire brigades, technical support in the form of helicopters and trucks, and predictive fire risk modelling, as well as environmental education programmes to dissuade indigenous and local people from burning. Critiques of widespread fire suppression policies underlined the unique role fire plays in the ecologies and cultures in many parts of the world, as well as highlighting the ineffectiveness of these policies (McDaniel et al. 2005; Sletto 2008; Sorrensen 2009; Carmenta et al. 2013; Mistry et al. 2016). This stimulated a turn in the tide as fire managers realized that a different approach was needed; one that addressed the continued occurrence of wildfires with the changing socio-economic situation of countries, the conflict of interests with local communities, and the emerging effects of climate change.

Indeed, after several decades of frustrated attempts to implement zero-fire policies, Brazil and Venezuela have, over the last 2–3 years, started to consider and implement fire management policies (Bilbao et al. 2010, 2017; Schmidt et al. 2016, 2018) (Box 1). These policies seek to reintroduce fire as a management tool in fire-prone ecosystems in order to (re)create seasonal mosaic landscapes, manage dry fuel and avoid large and catastrophic wildfires. This represents a major paradigm shift in fire management policies. In Brazil, prescribed early dry season fires, based on the Australian savannas experiences of valuation and reinterpretation of indigenous burning practices (Bliege Bird et al. 2008; McGregor et al. 2010; Russell-Smith et al. 2013, 2015), are an important aspect of the management techniques which aim to consider TEK and actively involve local communities. In Venezuela, the integration of indigenous burning practices with ecological knowledge from long-term collaborative fire experiments in savanna-forest gradients constituted the basis of a patch-mosaic burning model to be applied in Canaima National Park (Bilbao et al. 2006, 2009, 2010; Rodríguez et al. 2013a, b). However, while signifying major advances, as we discuss below these new fire management programmes need to be based on rigorous assessment of the local socio-ecological context in Brazil and Venezuela to ensure management goals are achieved. For example, the excessive concentration on early dry season fires to prevent late dry season fires may in fact affect the existence of landscape pyrodiversity and exclude local productive activities (Oliveira et al. 2015; Petty et al. 2015; Laris et al. 2016).

**Box 1:** Recent fire management developments in Brazil and Venezuela

Since 2014, Brazil and Venezuela have started to consider and implement fire management policies, through networks of research, expertise and international cooperation.
In Brazil, the Ministry of Environment, co-funded by the German Cooperation Agency and piloted in three large (> 150 000 ha) protected areas (PAs) initiated the Cerrado–Jalapão project. Located in the northern Cerrado (savanna), this Integrated Fire Management programme aims to: (i) change the predominant burning season in PAs, especially reducing the areas hit by late-dry season wildfires; (ii) protect fire-sensitive vegetation, such as riparian forests, from wildfires; (iii) enhance PA staff decision-making and fire management abilities, and; (iv) decrease conflicts between PA and local communities. The project has close links with the Australian savanna fire management model (Russell-Smith et al. 2013, 2015) and involves advice and exchanges between Australian and Brazilian park managers (Schmidt et al. 2018). Local research to determine management goals and fire regimes, and continuous evaluation will be essential to adapt international experiences to the Brazilian socio-ecological context.
In Venezuela, there has been a longer history of trying to move away from solely fire-fighting, focused in the Canaima National Park (CNP) in the south-east of the country. The CNP contains the headwaters of the Caroní River which supplies the Guri Reservoir where 70% of the country’s hydroelectric power is generated. Here, wildfires are a regular occurrence, and in spite of carrying out expensive and enormous fire suppression efforts, on average only 13% of total fires are combated (EDELCA-CORPOELEC 2008). A series of participatory action research projects funded by the national science financing agency (FONACIT) have brought together ancestral Pemón indigenous fire knowledge, scientific debate and inclusive dialogue between indigenous communities, fire-fighters, institutional and academic stakeholders about the socio-ecological issues of the CNP (Bilbao et al. 2010, 2017; Rodríguez et al. 2013a, b). Fire experiments initiated in 1999 for 11 years in savanna-forest gradients simulating traditional Pemón fire management techniques have shown how burning at different times during the dry season generate heterogeneous fuel patterns and biodiversity which reduce the risk of hazardous wildfires and protect the most vulnerable and diverse riparian and tropical humid forests (Bilbao et al. 2006, 2009, 2010).
In the past 2 years, the Brazilian and Venezuelan experiences have converged in several meetings and workshops, and we (the authors) have organized and facilitated multi-stakeholder meetings on fire management in Parupa, Venezuela (2015)^a^ and in Brasilia, Brazil (2017)^b^ involving local indigenous and traditional community representatives, scientists, environmental managers and government officials. These have contributed to the development of a national fire management policy in Brazil (currently at consultation phase with the explicit aim to include traditional fire practices and promote intercultural fire management) and the adoption of intercultural and participatory fire management by the Venezuelan government as part of their core policies and plans for the Venezuelan Protected Areas National System.

^a^See http://projectcobra.org/participatory-and-intercultural-fire-management-network

^b^See http://projectcobra.org/report-on-intercultural-and-participatory-fire-management

## Initial lessons learned

There are advances and challenges associated with the new fire management approaches in Brazil and Venezuela. Here, we point out some of the inherent tensions and barriers faced by fire managers.

This is the first time in Brazil and Venezuela that natural resource managers are actively planning and starting large-scale prescribed fires, a major step forward for conservation agencies (Rodríguez et al. 2013a, b; Millán 2015; Bilbao et al. 2017; Schmidt et al. 2016, 2018). Although there is a growing body of scientific knowledge on the effects of fire on Neotropical biodiversity (Durigan and Ratter 2016), not all species or situations have been studied. This is especially important when one considers the broadly applicable information fire managers might need or use to take management decisions (Driscoll et al. 2010). The inherent dynamic nature of fire means that predicting the outcomes of all actions is impossible, and a decision of no-action (not actively managing fire) is also a management decision with consequences.

In Brazil, for example, the past decades of ‘zero-fire’ policies in protected areas of the Cerrado (savanna) biome have commonly led to large (>50 000 ha) areas being consumed by wildfires in several hours or a few days (Barradas 2017). Similarly, Canaima National Park in Venezuela has been subject to increasingly larger fires, reaching 32 000 ha in a single dry season, fuelled by high accumulation of dry combustible materials (Bilbao et al. 2010). The human and financial resources mobilized to try to control such wildfires exceed several times the protected areas’ annual budget. The detrimental consequences of such wildfires should therefore be compared to the potential benefits of smaller, controlled fires started with the intention to create a burning mosaic that helps avoid wildfire propagation. For that, managers should be allowed to perform fire management considering uncertainty, and the fact that all species and/or effects will not be known in these highly diverse ecosystems.

Acknowledging that traditional groups from different localities have in-depth contextual knowledge on fire management (Mistry et al. 2005; Bilbao et al. 2010; Welch 2015; Eloy et al. 2017), new fire management policies in Brazil and Venezuela are attempting to incorporate TEK into their processes and techniques. In Brazil, for example, elders from local communities are engaged to produce fire calendars that form part of the prescribed burning plans. In some instances, where the traditional practices of fire management were lost, for example in the Indigenous territory of the Xerente, Brazil, institutions are ‘rescuing’ TEK to reapply it for conservation purposes (Falleiro et al. 2016). A national fire management policy currently being drafted in Brazil aims to explicitly include TEK and its adaptive capacity to address current and future environmental challenges. In Venezuela, the indigenous Pemón communities of Canaima National Park have been involved in joint ecological experiments as a process of strengthening and regaining fire TEK, as well as consulting and learning from elders on fire calendars and ancestral practices. Improved dialogue between communities and institutions has led to a greater receptiveness by the Pemón to exchange and share their knowledge. The new fire management plan for the Park will consider both traditional, technical and scientific knowledge to decide where, when and how to set fires, as well as include formal agreements between communities, EDELCA, INPARQUES and the Ministry of Science and Technology (Bilbao et al. 2017).

These developments in fire policy and associated programmes are significant, and government institutional advocacy for greater intercultural and participatory fire management must be recognized. At the same time, further improvements in the process of involving traditional communities could lead to better outcomes for all. At the multi-stakeholder meeting on fire management in Brasilia in 2017, we (the authors) asked the indigenous and traditional community representatives, scientists, environmental managers and government officials, to reflect on the following: What is participation? How is this viewed and implemented by different actors? How could the formation of official brigades affect the dynamics of collective fire management in the communities? Who makes the decisions? How can conservation institutions and local communities interact to improve fire governance? How can fire management be a community owned solution? How can fire management be integrated into people’s everyday activities and livelihoods? How can indigenous and scientific knowledge work together for more effective fire management?

In the current policies, local community meetings are central to the fire management programmes. However, staff from environmental institutions are not trained nor used to consider TEK to define or apply environmental policies, exacerbated by the perception that TEK is something of the past, static, without technical value and not responsive to current and future challenges. In parallel, local communities have no valid reason to believe or collaborate with institutions that have marginalized their knowledge and practices for so long. Therefore, when these meetings are performed, participation seems to be more of a ‘consultation’ where TEK is seen as a source of information that can be incorporated into institutionalized processes, thus (re)establishing hierarchical relationships where environmental managers’ technical decisions are worth more than local peoples’ opinions.^1^

This can be made worse by the increasing dependency on geospatial technologies and global science metrics (emissions) (Sletto 2008; Mistry and Bizerril 2011; Carmenta et al. 2013). In the well-documented Australian case, large-scale burning often implemented by helicopters and technicians, increased a sense of disengagement of Aboriginal people from their territory (Eriksen and Hankins 2014; Fache and Moizo 2015; Petty et al. 2015; Perry et al. 2018). Furthermore, to date, local participation in prescribed burning schemes has come mostly in the form of professionalized, and to some extent, militarized, rangers/brigades. Brazil, for example, has invested in ‘community-run’ brigades since the mid-2000s. Although these fire brigades are used as a way to ‘integrate’ TEK and scientific knowledge about fire management, the technical training and the fact that people are hired specifically to manage fire could move practices away from collective governance (a norm in many traditional communities) to individual actions, discouraging members of the wider community from taking responsibility for wise fire management and maintaining the subordination of local practices to those of external experts (Mistry et al. 2016).

As seen in the Australian case, institutionalized fire management programmes risk turning local communities to beneficiaries of a service, rather than promoting self-determination and responsibility for the management of the land they live in (Eloy et al. 2016, 2017). With a focus on early dry season burning to protect against late dry season wildfires, the policies fail to recognize that traditional fire management is characterized by multiple, and sometimes opportunistic, burning throughout the year linked to various social, ecological and spiritual purposes, which produce the mosaic landscapes to help buffer the impacts of climate variability and maintain biodiversity (Bilbao et al. 2009, 2010; Laris et al. 2016). In addition, incorporating local uses of fire for productive activities such as swidden agriculture and livestock grazing can represent a challenge, since these fires frequently depend on late dry season fires which are generally perceived as ‘bad’ fires (Eloy et al. 2018).

**Figure 1:**
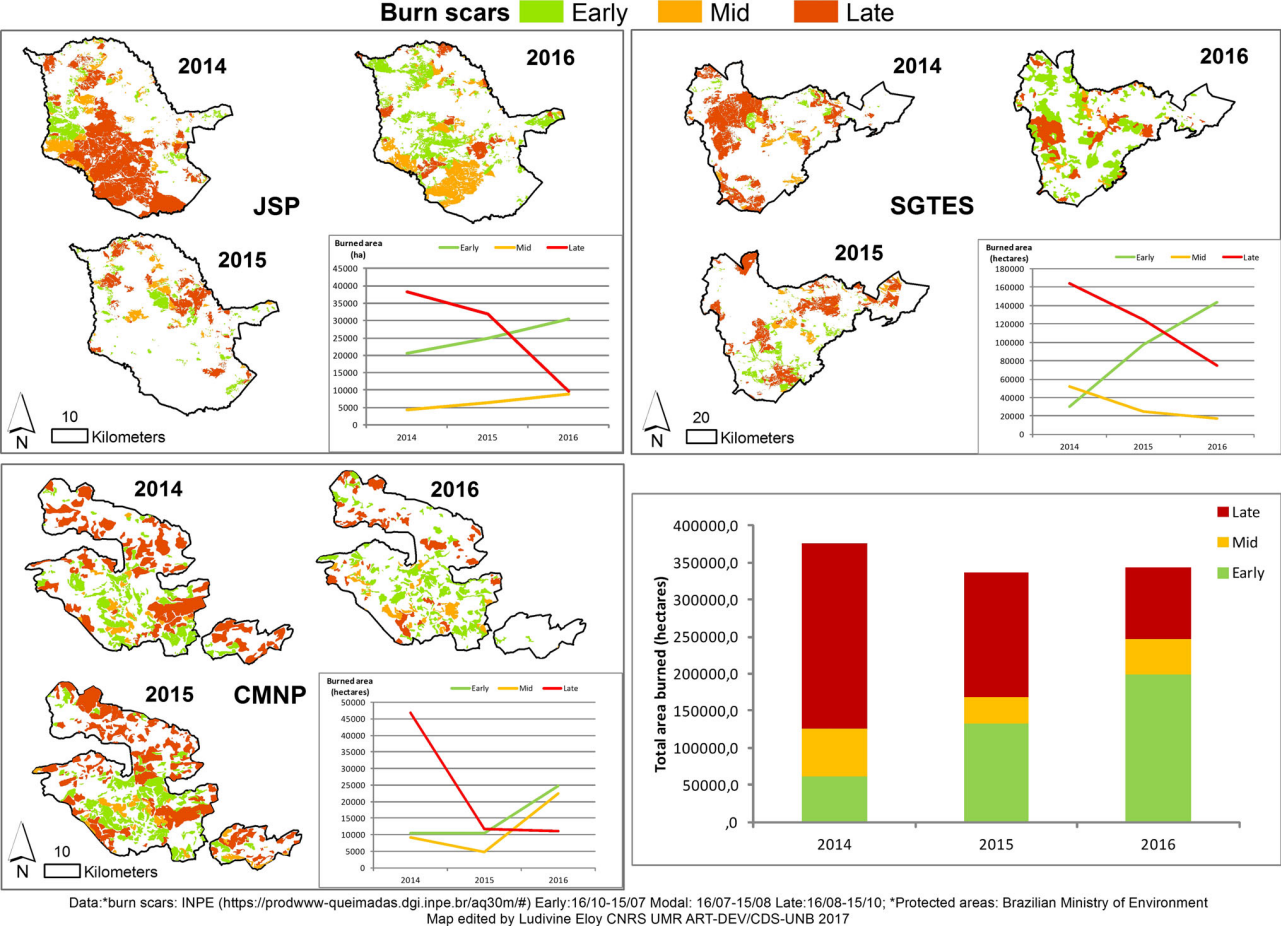
Maps of burn scars according to fire season in the three protected areas of IFM implementation in the Brazilian savanna from 2014 to 2016. *JSP* Jalapao State Park, *SGTES* Serra Geral do Tocantins Ecological Station, *CMNP* Chapada das Mesas National Park. Prepared by Ludivine Eloy (we used burn scars data from the Brazilian Institute of Space Research (INPE) (https://prodwww-queimadas.dgi.inpe.br/aq30m/), with a 30-m resolution produced from Landsat imagery. Using ArcGIS software, we compiled all the shapes of burn scars from 2014 to 2016, dividing data between three periods: early, modal and late, with at least three sets of data per period (early dry season: 16th October–15th July; mid dry season: 16th July–15th August; late dry season: 16th September–15th October). We adopted ICMBio’s periods and classification for fire seasons)

Reflecting on Aboriginal fire management in northern Australia, Petty et al. (2015) suggest that “it is inherent in the nature of institutionalized management programs to replace the complexity and contingency of indigenous fire management with standardized goals” (p. 140). We see this happening in Brazil.^2^ Preliminary evidence from the Integrated Fire Management (IFM) programme in Brazil shows a small decrease in total burned area, but a significant reduction in the percentage of late dry season emissions, which is one of the main goals of the programme in the three protected areas (Fig. 1). Since emissions from fires account for 28% of land use emissions, this reduction is now strategic for the Brazilian government and included in its 2016 National Emission Inventory. However, there is considerable uncertainty on the impacts of early dry season burning on fire intensity and biodiversity (Oliveira et al. 2015; Laris et al. 2016). Long-term experiments from the Gran Sabana, Venezuela have shown a higher daily variability in fire behaviour associated with weather conditions, fine fuel load and wind velocity, compared to along the dry season (Bilbao et al. 2006, 2009, 2010). Likewise, the general pattern of plant cover and biomass recovering from pre-fire conditions revealed higher and faster rates from middle dry season burns compared to early and late burns (Bilbao et al. 2009). A switch, therefore, from late to early dry season burning requires much greater local level assessments of above ground biomass, burn severity, fuel burn completeness, and GHG emissions in order to provide evidence for its efficacy towards improving savanna management and supporting local productive activities.

Achieving emissions reductions goals has led to a narrative of and investment in ‘alternatives to the use of fire’ within the IFM programme. This is justified by arguments that traditional fire knowledge has been or soon will be lost so other solutions are needed, that fire-free methods are more ‘modern’, productive and a way out of poverty, and that carbon emissions from agriculture and grazing could be reduced by fire-free farming and grazing techniques. However, these approaches can only reinforce the idea that traditional uses of fires are obsolete, indicating that advancing fire management policies requires not only technical and ecological information, but also much more work on changing preconceptions and the dominant institutional discourses about fire use.

## Towards better intercultural governance

Recent meetings in Parupa, Venezuela and in Brasilia, Brazil facilitated by the authors and involving local community representatives, scientists, fire/environmental managers and government officials, have shown the importance of bridging local, technical and scientific understandings of fire and its governance (Rodríguez et al. 2013a, b; Mistry and Berardi 2016). These events have allowed collaborative and reflective dialogue on policy and practice, an opportunity for learning across different communities, as well as between communities and institutions. We argue that supporting processes for integrating multiple perspectives through an ‘intercultural interface’ of institutions and knowledge systems (Goldman et al. 2011; Howitt et al. 2013; Tengö et al. 2014) is critical as Brazil and Venezuela transition towards more participatory forms of fire management and governance. This can be done through:

- training decision-makers and PA managers in participatory methods that encourage engagement with, and appreciation of, indigenous and traditional perspectives and practices of fire management. For example, in a recent workshop focused on the management of Canaima National Park, we facilitated training for scientists and government agencies on participatory video and community owned solutions approaches to working with indigenous communities.^3^
- legitimizing and strengthening indigenous and traditional fire management as a community owned solution grounded in local social–ecological systems. For example, promoting regional participatory workshops and field experiments could help understand fire behaviour, fire propagation and local productive fire uses, and how they could be more effectively included in fire management programmes. We are promoting this in the Jalapão savanna region regarding the burning of fire-sensitive wet grasslands. These areas are simultaneously targeted for fire management by local communities for plant harvesting and cattle raising, and by landscape managers for protecting fire-sensitive riparian forests. Finding common fire management practices of these wet grasslands can improve productive practices, conserve biodiversity and reduce conflicts.
- creating spaces for continual multi-stakeholder conversations about fire management, where different perspectives and experiences can be shared, and where action plans to improve fire management can be co-developed. Actions have to be aimed at encouraging indigenous and traditional communities more autonomy with respect to implementing policies, including the leadership and funding of fire management programmes. In Venezuela, a plan for joint training between the Pemón indigenous community of Kavanayén, Canaima National Park and Forest Firefighters of INPARQUES is underway. Elders of the Kavanayén community will share their knowledge and train forest firefighters on ancestral practices, and in turn firefighters will train young Pemón on fire combat techniques used to control accidental wildfires. Prescribed fires will be jointly planned, implemented and evaluated, and indigenous representatives hope to share their experiences with other indigenous communities in the park.

Brazil and Venezuela, two countries where fire management has been highly contested, have undergone a major paradigm shift in their approaches to fire management. Despite the progressive nature of these policies, it is critical that we build a collective adaptive learning environment in which we can experiment and monitor fire management methods and interventions while giving an equal footing to scientific and local knowledge as valid systems of information that can be used for fire governance. Only by working hand in hand, can we prevent frequent catastrophic wildfires and maintain local communities’ livelihoods and cultures that help to protect highly threatened fire-prone ecosystems.

## Footnotes

1 However, note that in Venezuela, over the past few years, there has been significant commitment and understanding from the Forest Firefighters of INPARQUES about the role and importance of TEK, and they have encouraged and promoted full participation of the indigenous Pemón in fire management.

2 The following observations are derived from presentations made at the Cerrado-Jalapão project meeting in Brasilia in November 2017.

3 See http://projectcobra.org/learning-and-planning-together-for-action-on-climate-change-mitigation-and-adaptation.
